# Patient Preferences for Nonvitamin K Antagonist Oral Anticoagulants in Stroke Prevention: A Multicountry Discrete Choice Experiment

**DOI:** 10.1155/2019/5719624

**Published:** 2019-12-18

**Authors:** Thomas Wilke, Anna-Katharina Meinecke, Bernhard Schaefer, Sandra Buchwald, Daniel Eriksson, Sabrina Müller

**Affiliations:** ^1^Ingress-Health HWM GmbH, 23966 Wismar, Germany; ^2^Bayer AG, 13342 Berlin, Germany

## Abstract

**Purpose:**

The patient's perspective is becoming increasingly important in clinical and policy decisions. This study examined atrial fibrillation (AF) patient preferences for different characteristics of nonvitamin K antagonist oral anticoagulants (NOACs).

**Methods:**

A discrete choice experiment (DCE) addressing AF patients treated with NOACs in France, Germany, and the United Kingdom was conducted. The DCE included the following attributes: frequency of administration (once/twice daily), size of tablet/capsule (6–9 mm/20 mm), meal-related intake (intake with food required/independent), and distance to treating physician (1 km/10 km). Preferences were analyzed based on a conditional logit regression model.

**Results:**

In total, 758 patients (males: 57.3%; mean age: 71.4 years) with an average disease duration of 5.5 years were included (apixaban/dabigatran/edoxaban/rivaroxaban: 34.0%/14.5%/6.6%/44.9%, respectively). Patients preferred NOAC treatment options characterized by once-daily dosing regimens (42.8%; *p* < 0.001), shorter distance to treating physicians (25.0%; *p* < 0.001), a small-sized tablet (21.5%; *p* < 0.001), and intake independent of food (10.6%; *p* < 0.001).

**Conclusions:**

Patients primarily prefer a once-daily NOAC regimen. Individual preferences should be considered for the treatment of AF patients as this may result in improved treatment adherence and consequently better effectiveness and safety in routine clinical practice.

## 1. Rationale and Background

Atrial fibrillation (AF) is the most commonly sustained cardiac arrhythmia. It is estimated to occur in around 3% of adults aged 20 years and older, with a higher prevalence in the elderly and in patients with comorbidities such as hypertension, coronary artery disease, diabetes mellitus, and chronic kidney disease [1]. AF is an independent risk factor for stroke, heart failure, and mortality [2, 3] and increases the risk of stroke approximately five folds [3].

For many decades, warfarin and other vitamin K antagonists (VKAs) have been the mainstay of stroke prevention therapy in AF patients. In recent years, a number of non-VKA oral anticoagulants (NOACs) have been introduced to the market. In terms of efficacy and safety, NOACs have been shown to be at least as effective and safe as VKAs for stroke prevention in patients with nonvalvular AF (NVAF) [4–9]. However, the lack of head-to-head trials of NOACs creates a challenge for physicians and patients in the choice of anticoagulation therapy.

Patient involvement and individual preferences are highlighted in treatment guidelines as important factors in the initiation of treatment and long-term management of AF patients [1, 10]. Even though previous research has shown that AF patients prefer once-daily anticoagulation dosing [11, 12] therapy that does not require periprocedural bridging of anticoagulation and anticoagulation therapy that does not interact with food [12], here is only limited literature on patient preferences regarding different NOACs available. The objective of this study was to elucidate AF patients' preferences for attributes associated with NOAC therapy.

## 2. Research Methods

This was a cross-sectional study of AF patients in France, Germany, and the United Kingdom (UK). Study inclusion criteria were at least 18 years of age; diagnosis of AF; treated with an NOAC (apixaban, dabigatran, edoxaban, or rivaroxaban) for at least the past three months; and willing to be interviewed over telephone. The structured interviews were computer-assisted and conducted by trained interviewers.

We conducted a discrete choice experiment (DCE) to measure and quantify patient preferences for NOAC therapy in AF. The DCE is a well-accepted approach for patient preference measurement in health care. Choice experiments examine preferences using pairwise comparisons of holistic hypothetical alternatives instead of ranking or assessing single features only [13, 14]. A series of evaluation tasks are presented to the respondent who needs to select one of the presented alternatives in each task. Each alternative is described by predefined attributes that vary across the different alternatives. The variation across the alternatives in the choice sets is achieved by assigning different levels to the attributes. The basic assumption of a DCE is that rational individuals will always choose the alternative with the higher level of expected utility. In this way, the degree to which each attribute (treatment characteristic) influences the choice of the patient can be examined [15, 16].

The study included treatment characteristics identified in the literature as influencing anticoagulation therapy preferences [12] and NOAC-related factors identified in the European summaries of product characteristics for each respective anticoagulant [17–20]. Due to the lack of head-to-head randomized trials of NOACs, there is no clinical evidence of differences between the medications in terms of efficacy and safety. Consequently, these attributes were not included in the study. In addition, a comparator attribute defined as distance to the treating physician was included as a way to express the marginal valuations of the treatment attributes in terms of an easily understood unit (distance in kilometer (km)).  Table 1 provides a summary of the included attributes and their levels as used in the DCE.

A fractional factorial design was generated using IBM SPSS Statistic software (v 20) [21, 22]. Based on an orthogonal main-effects design, a set of eight different choice sets, each with two alternative treatment options was derived. A ninth choice set was integrated to assess consistency of response behavior. This test set duplicated a previous decision situation with interchanged treatment options. All choice sets were graphically visualized to facilitate comprehension of the different attributes and their levels (see Supplementary [Supplementary-material supplementary-material-1] for an example choice set used in the study).

Baseline patient characteristics were analyzed using descriptive statistics. Categorical data were reported as proportions and continuous data as mean, standard deviation, and median. Patients with inconsistent DCE data were excluded from the analyses. Inconsistency was assessed based on responses on the abovementioned test set. The DCE dataset was analyzed using a conditional logit regression model that included all attributes (NOAC treatment characteristics) as independent variables. The conditional logit relates the probability of choice among the alternatives (choice sets) to the characteristics of the attribute levels defining those alternatives [15]. The relative importance of the NOAC treatment attributes was estimated for the overall sample and for prespecified subgroups.

## 3. Main Results

A total of 898 patients completed the study. Of these, 140 (16%) were excluded due to inconsistency in their responses, resulting in a study population of 758 patients (Germany: 280 (37%); France: 338 (45%); UK: 140 (18%)). Median age was 72 years, and 43% were female. On average, patients had been diagnosed with AF more than 5 years ago and been on treatment with their current NOAC for 2 years. Overall, 34% of patients were treated with apixaban, 14% with dabigatran, 7% with edoxaban, and 45% with rivaroxaban. A comprehensive list of baseline characteristics is provided in Table 2. As also shown in Table 2, patients who were excluded due to inconsistent response behavior were significantly older, but were otherwise similar to the study population in terms of demographic and clinical characteristics.

Table 3 shows the results of the conditional logistic regression analyses. A once-daily dosing regime was strongly preferred in comparison with twice-daily dosing (utility: 0.80; *p* < 0.001). Patients also expressed preference for a tablet sized 6–9 mm rather than a 20 mm capsule (utility: 0.40; *p* < 0.001) and a medication that does not need to be taken with food (utility: 0.20; *p* < 0.001). Patients also favored a shorter distance to the treating physician (utility: 0.47; *p* < 0.001).

**Figure 1:**
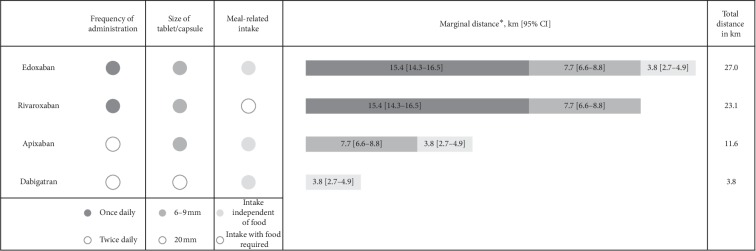
Derived willingness to accept additional distance to the treating physician for different available NOACs.

The marginal valuations of the treatment attributes (expressed as distance willing to travel for an improvement in a positively valued attribute) are presented in Table 3 as well as in Figure 1. On average, patients were willing to travel an additional 27 km to their treating physician in order to receive an NOAC that combines all the preferred attribute levels (once-daily dosing, small tablet size, and a medication that does not need to be taken with food). For a treatment option that provides at least a once-daily dosing (instead of twice-daily), patients were willing to accept a longer distance of 15.4 km. The equivalent distances for receiving a smaller tablet and a medication that can be taken with or without food were 7.7 km and 3.8 km, respectively.

An overview of the relative impact of each of the four attributes stratified by patient characteristics is provided in Supplementary [Supplementary-material supplementary-material-1]. Preferences were generally similar across subgroups with once-daily dosing frequency valued the highest.

## 4. Discussion

Our study adds new evidence on patient preferences for NOACs. The results of our study illustrate that patients prefer an NOAC regimen consisting of a single daily dose. Frequency of administration was the most important attribute (42.8% impact on the overall decision of patients), followed by distance to treating physician (25.0%) and size of the tablet/capsule (21.5%). The least important attribute was meal-related intake (10.6%).

One of the advantages of the DCE methodology is that properties of specific treatments (attribute levels) can be transferred into utilities that in turn describe trade-off patients make in deciding between treatment options. Compared with the (theoretically) least preferred NOAC treatment option, the highest utility (expressed as accepted distance to the treating physician) was observed for the once-daily treatment options edoxaban (27 km) and rivaroxaban (23.1 km), followed by apixaban (11.6 km) and dabigatran (3.8 km) as the least preferred NOAC treatment options. Our findings were generally consistent across subgroups of patients with a once-daily dosing frequency preferred over twice-daily administration. While still ranking once-daily dosing as the most important attribute, the elderly and women seemed to put more relative value on having a shorter distance to their treating physicians compared with the general AF population. Furthermore, relative to the study population, patients in France seemed to value a treatment that can be taken independently of food, while this attribute was of minimal importance to patients in the UK. Based on analyses by treatment, the data seemed to indicate a level of adaption by patients to the current NOAC regimen, which may be explained by the development routines for taking medications long-term [23]. For example, patients receiving apixaban, dabigatran, or rivaroxaban seemed to rank attributes specific to these treatments relatively low (dosing frequency, capsule size, and meal-related intake, respectively; see Supplementary [Supplementary-material supplementary-material-1]).

Our findings are consistent with previous preference studies. In a European survey of 1,507 patients with AF, 80.7% of patients expressed a preference for taking anticoagulation medication once daily compared with only 7.6% who preferred a twice-daily regimen [11]. Twice-daily dosing frequency was also identified as a barrier to treatment adherence in an Italian study of 525 patients treated with VKAs who were assessed for switching to NOACs [24]. In another preference study conducted in Germany, Switzerland, and Sweden using a DCE approach, frequency of administration also prevailed as the most important treatment attribute among other regimen-related characteristics comparing VKAs and NOACs [25].

We acknowledge some limitations of our study. Selection bias arising from differences in patient characteristics between those willing to participate in the study and those who declined to participate cannot be ruled out. The information presented to patients in the DCE is a simplification of reality, and it is possible that unobserved attributes influenced the decisions of patients. While the DCE can be perceived as a complex approach for both interviewers and patients, the interviewers in our study were intensively trained and supported by a guideline on how to design a hypothetical atmosphere within the experiment. Furthermore, the choice situations were visualized and made available to participants.

## 5. Conclusions

This study showed that patients primarily prefer a once-daily NOAC regimen. Individual preferences should be considered for the initiation and long-term treatment of AF patients as this may result in improved treatment adherence and consequently better effectiveness and safety in routine clinical practice.

## Figures and Tables

**Table 1:** Attributes and levels used in the discrete choice experiment.

Attribute	Level 1	Level 2
Frequency of administration	Once daily	Twice daily
Size of tablet/capsule	6–9 mm tablet	20 mm capsule
Meal-related intake	Intake with food required	Intake independent of food
Distance to treating physician	1 km	10 km

**Table 2:** Patient characteristics.

Variable		Patients included	Patients excluded due to inconsistent DCE data
*N*		758	140
Age in years, median, mean (SD)		72, 71.4 (9.9)	77, 74.4 (10.0)^*∗*^
Female gender, *n* (%)		324 (42.7)	60 (42.9)
BMI, median, mean (SD)			
Male		27, 28.1 (4.7)	27, 27.8 (5.8)
Female		26, 27.5 (5.6)	26, 26.5 (5.5)
Country, *n* (%)			
Germany		280 (36.9)	42 (30.0)
France		338 (44.6)	62 (44.3)
UK		140 (18.5)	36 (25.7)
AF duration in years, median, mean (SD)		3, 5.5 (6.4)	4, 5.7 (6.1)
Duration of current NOAC treatment in years, median, mean (SD)		2, 2.1 (1.7)	2, 2.4 (2.0)
Current anticoagulant, *n* (%)			
Apixaban	2.5 mg	73 (9.6)	17 (12.1)
	5 mg	185 (24.4)	32 (22.9)
Edoxaban	30 mg	11 (1.5)	1 (0.7)
	60 mg	39 (5.2)	4 (2.9)
Dabigatran	110 mg	50 (6.6)	8 (5.7)
	150 mg	60 (7.9)	8 (5.7)
Rivaroxaban	15 mg	97 (12.8)	21 (15.0)
	20 mg	243 (32.1)	49 (35.0)
Frequency of administration, *n* (%)			
Apixaban	Once daily	2 (0.3)	0 (0.0)
	Twice daily	256 (33.8)	49 (35.0)
Edoxaban	Once daily	50 (6.6)	5 (3.6)
	Twice daily	0 (0.0)	0 (0.0)
Dabigatran	Once daily	2 (0.3)	0 (0.0)
	Twice daily	108 (14.2)	16 (11.4)
Rivaroxaban	Once daily	340 (44.9)	70 (50.0)
	Once daily	0 (0.0)	0 (0.0)
Patients previously prescribed with OAC, *n* (%)		50 (6.6)	9 (6.4)
Currently taking other prescription medications regularly, *n* (%)		720 (95.0)	134 (95.7)
Number of different medications, median, mean (SD)		3, 4.0 (2.9)	4, 4.6 (2.9)
Comorbidities, *n* (%)			
Heart failure		224 (32.2)	53 (37.9)
Hypertension		472 (62.3)	75 (53.6)
Diabetes		166 (21.9)	31 (22.1)
Stroke		75 (9.9)	20 (14.3)
Impaired kidney function		65 (8.6)	15 (10.7)
Vascular disease		95 (12.5)	23 (16.4)
Type of primarily treating physician, *n* (%)			
GP		220 (29.0)	51 (36.4)
Cardiologist		627 (82.7)	110 (78.6)
Others		27 (3.6)	2 (1.4)

^*∗*^statistically significant at *p* < 0.05.

**Table 3:** Results of the conditional logit model.

Attribute	Levels	Full model (fixed effect)		
		Coefficient	*p* value	95% CI	Marginal distance^*∗*^, km [95% CI]
Frequency of administration	Once daily	0.795	<0.001	[0.738–0.853]	15.4 [14.3–16.5]
	Twice daily (reference)				
Size of tablet/capsule	Tablet 6–9 mm	0.400	<0.001	[0.343–0.456]	7.7 [6.6–8.8]
	Capsule∼20 mm (reference)	0			
Meal-related intake	Intake independent of food	0.198	<0.001	[0.142–0.254]	3.8 [2.7–4.9]
	Intake with food required (reference)	0			
Distance to treating physician	1 km	0.465	<0.001	[0.408–0.522]	n/a
	10 km (ref.)	0			

^*∗*^The marginal estimate of the distance that an average patient is willing to travel to the treating physician in order to obtain the specified attribute level compared with the reference level.

## Data Availability

The data supporting the findings of this study are available from the corresponding author (SM) upon reasonable request.

## Additional Points

*Key Points.* (i) This cross-sectional observational study assessed NOAC treatment preferences of AF patients in France, Germany, and the United Kingdom, using a discrete choice experiment framework; (ii) patients preferred NOAC regimens with once-daily dosing frequency and tablets/capsules of smaller size; (iii) patients were willing to accept a significant burden in order to receive their preferred NOAC treatment; they were willing to travel an additional 27 km to their treating physician in order to receive a NOAC with all the preferred attributes

## Supplementary Materials

Supplementary Materials Supplementary Figure 1: example of a visualized DCE set. Supplementary Figure 2: relative importance of NOAC treatment attributes.
