# Supplementary material for: Patient Preferences for Nonvitamin K Antagonist Oral Anticoagulants in Stroke Prevention: A Multicountry Discrete Choice Experiment
Source: Cardiol Res Pract. 2019 Dec 18;2019:5719624. doi: 10.1155/2019/5719624 (PMC6935812; doi:10.1155/2019/5719624)
Supplement: Supplementary Materials — Supplementary Figure 1: example of a visualized DCE set. Supplementary Figure 2: relative importance of NOAC treatment attributes. [file 5719624.f1.pdf]

Supplementary Materials

Supplemental Figure 1: Example of a visualized DCE set

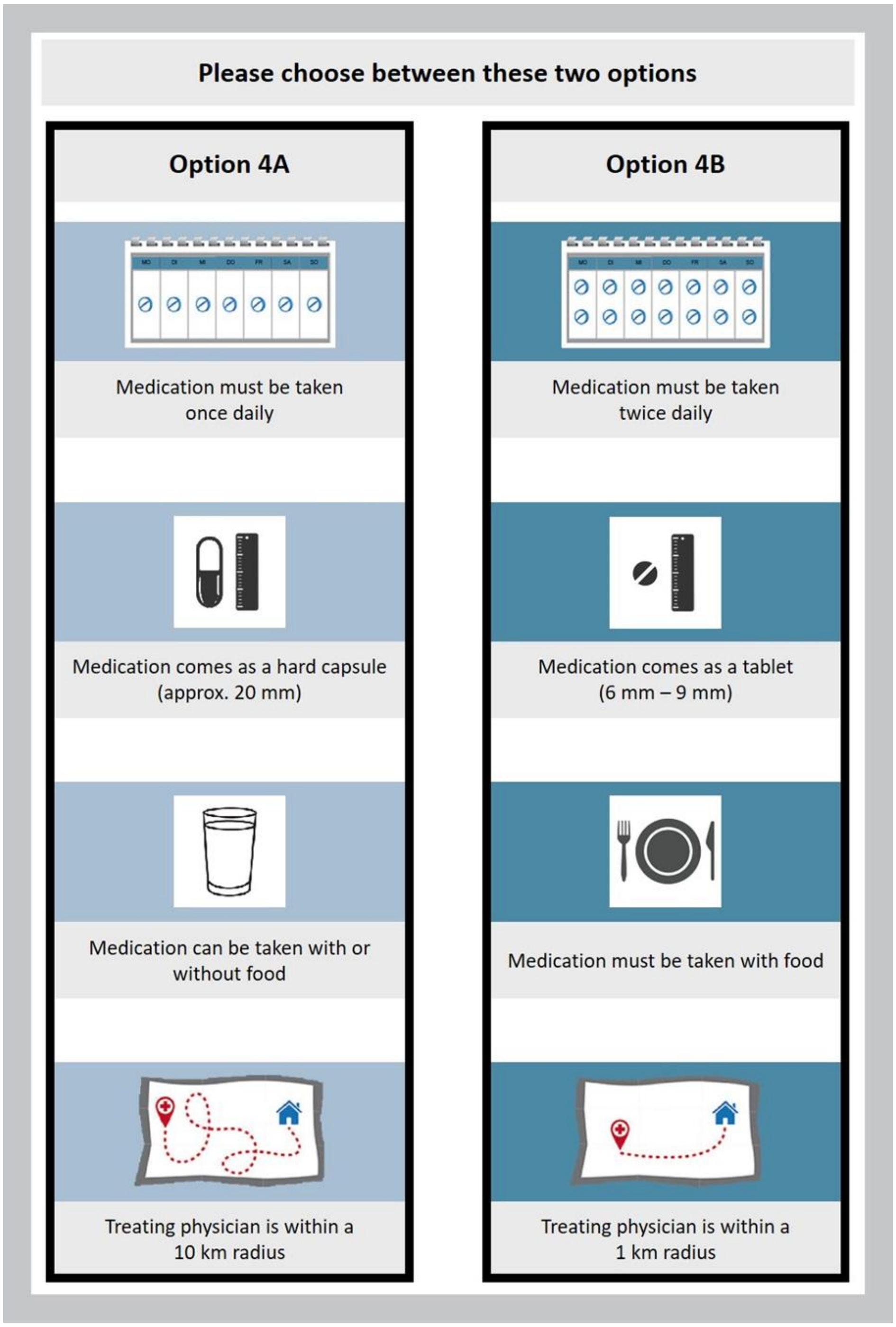

Example of visualized applied choice sets. The DCE card shows two hypothetical NOAC alternatives derived from a combination of the defined attributes and their respective levels. Patients were required to decide between option A or B (no opt-out).

Supplemental Figure 2: Relative importance of NOAC treatment attributes

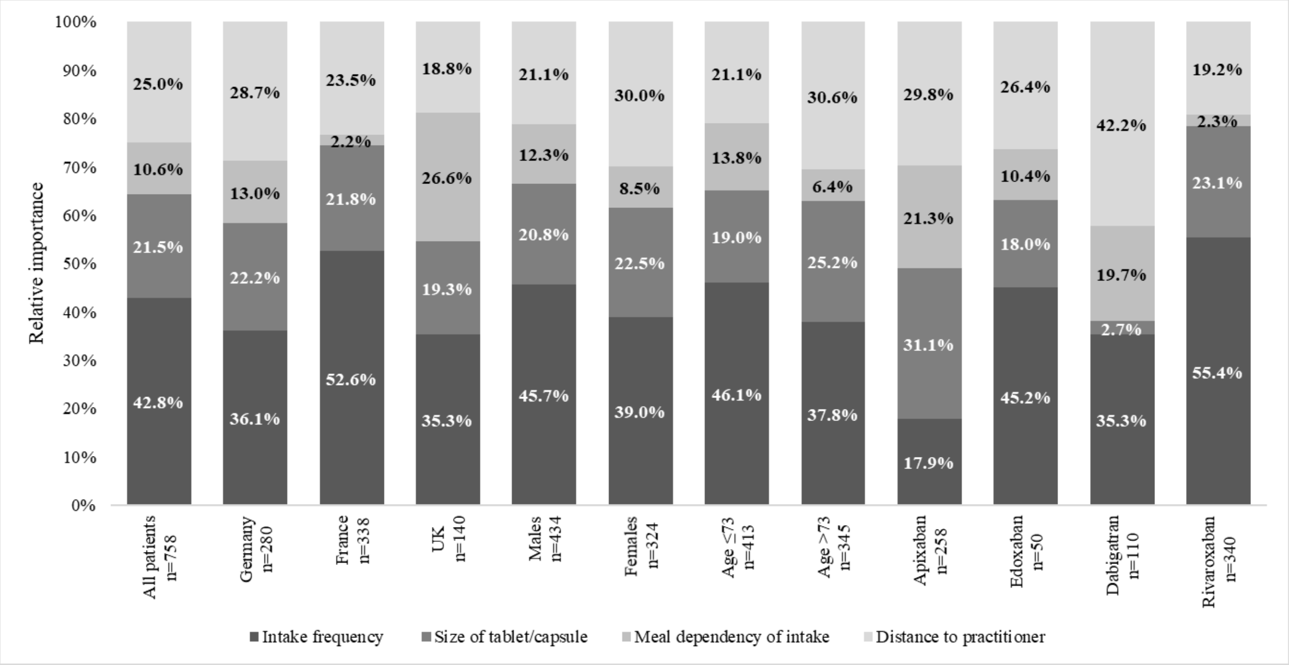

The figure shows the the relative importance of NOAC treatment attributes, estimated for the overall sample and for pre-specified subgroups. The DCE data were analyzed using logistic regression.
